# Propolis ethanolic extracts reduce adenosine diphosphate induced platelet aggregation determined on whole blood

**DOI:** 10.1186/s12937-018-0361-y

**Published:** 2018-05-14

**Authors:** Mirza Bojić, Andrea Antolić, Maja Tomičić, Željko Debeljak, Željan Maleš

**Affiliations:** 1 Faculty of Pharmacy and Biochemistry, Department of Medicinal Chemistry, University of Zagreb, A. Kovačića 1, 10000 Zagreb, Croatia; 2 Faculty of Science, PDS Biology, University of Zagreb, Rooseveltov trg 6, 10000 Zagreb, Croatia; 3 Department of Platelet and Leukocyte Immunology, Croatian Institute of Transfusion Medicine, Petrova 6, 10000 Zagreb, Croatia; 4 Institute of Clinical Laboratory Diagnostics, Osijek University Hospital Center, Josipa Huttlera 4, 31000 Osijek, Croatia; 5 Faculty of Medicine, University J. J. Strossmayer of Osijek, Josipa Huttlera 4, 31000 Osijek, Croatia; 6 Faculty of Pharmacy and Biochemistry, Department of Pharmaceutical Botany, University of Zagreb, Schrottova 39, 10000 Zagreb, Croatia

**Keywords:** Propolis, Flavonoids, Phenolic acids, Platelet aggregation, Thrombus formation, Cardiovascular diseases

## Abstract

**Background:**

Propolis is a well-known bee product containing more than 2000 identified compounds. It has many beneficial effects on human health that include antibacterial, antiviral, anticancer and hepatoprotective justifying its use as a dietary supplement. Platelet aggregation plays crucial role in thrombus formation that can cause stroke or heart attacks. As cardiovascular diseases, including those caused by thrombus formation, are related to 50% of deaths of Western population, the objective of this study was to determine antiaggregatory activity of propolis on platelet aggregation on the whole blood samples.

**Methods:**

Twenty one propolis samples from Southeast Europe were characterized by spectrophotometric methods to determine content of the total flavonoids and phenolic acids. High performance liquid chromatography coupled with diode array detection was used to identify and quantify individual polyphenols. Platelet aggregation was tested by impedance aggregometry on the whole blood samples of ten healthy volunteers.

**Results:**

The mean content of total polyphenols was 136.14 mg/g and ranged from 59.23 to 277.39 mg/g. Content of total flavonoids ranged between 6.83 and 55.44 mg/g with the mean value of 19.28 mg/g. Percentage of total phenolic acids was in the range 8.79 to 45.67% (mean 26.63%). Minimal antiaggregatory concentration, representing the lowest concentration of propolis extract sample that can cause statistically significant reduction of aggregation, ranged from 5 μM to 10.4 mM. Samples of propolis with lower content of luteolin and higher content of pinocembrin-7-methyleter showed better antiplatelet activity i.e. lower values of minimal antiaggregatory concentration.

**Conclusions:**

This is the first study that shows antiaggregatory potential of propolis ethanolic extracts on the whole blood samples in the low micromolar concentrations suggesting that propolis supplementation may influence platelet aggregation and consequently thrombus formation. Further in vivo studies are needed to confirm the beneficial effects in prevention of cardiovascular diseases.

## Background

Propolis is a well-known bee produced wax mixture containing more than 2000 identified compounds [1]. It is collected from by honeybees (*Apis mellifera* L.) from plants, primarily from flowers and leaf buds [2]. Chemical composition of this resinous mixture varies depending of the origin and plant species but in principle contains up to 45–55% polyphenols (flavonoids, phenolic acids and it esters), 25–35% beeswaxes (waxes and fatty acids), 10% volatile substances (essential oils), 5% pollen containing amino acids and 5% of other substances like minerals, vitamins and sugars [2].

Propolis has many beneficial effects on human health that justify its use as a dietary supplement. It is mainly used due to its antioxidant properties and along with wine represents the most significant source of polyphenols in Western world [3]. Many in vitro and in vivo animal studies have shown antibacterial [4], antiviral [1], hepatoprotective [5], immunomodulatory [6], anticancer [7] and other biological effects of propolis. These positive effects are primarily related to the content of flavonoids and phenolic acids [5]. Our group has extensively studied Croatian and other European propolis and found that propolis ethanolic extracts possess antibacterial activity against *Staphylococcus aureus* and antifungal activity against yeast *Candida albicans* [4]. Antioxidant activity assessed using diphenylpicrylhydrazyl, a colored free stable radical, was related to the content of *p*-coumaric acid, tectochrysin, and kaempferol [4]. The content of tectochrysin, galangin, pinocembrin chrysin, apigenin, and kaempferol was significantly correlated with antimicrobial activity while p-coumaric acid, apigenin, and kaempferol were significantly correlated with the activity of propolis against *C. albicans* [5]. Antitumoral activity against HeLa cell lines was related to the high concentrations of ferulic and *p*-coumaric acid, galangin and pinocembrin [8].

Cardiovascular diseases are related to 50% of deaths of Western population [9]. Direct and indirect costs of cardiovascular diseases are estimated to total more than $316 billion [9]. As people get more conscientious of their wellbeing and health protection, many opt for dietary supplements containing functional foods and nutraceuticals. One of the approaches is to prevent platelet aggregation i.e. primary step in blood clot (thrombus) formation that can cause heart attack [10], brain stroke [11], pulmonary embolism [12] and deep-vein thrombosis [13]. For this purpose, aspirin (acetylsalicylic acid) is often used, but as medicines are accompanied with significant side effects new regulators of platelet aggregation are of interest [14]. It has been shown that flavonoids [15] and phenolic acids [16] can prevent platelet aggregation.

By now, there have been two reports of antiaggregatory effect of propolis. Zhang et al. [17] tested platelet aggregation on platelet rich plasma and our group conducted preliminary research on whole blood [18]. However, in both studies concentration (dry mass expressed as molar equivalents of quercetin per volume of extract) that caused statistically significant reduction of aggregation induced by adenosine diphosphate were above 30 μM. As maximal concentration of flavonoids that can be found in blood goes up to 7.6 μM [19], further studies are needed to determine minimal concentrations at which propolis extracts prevent platelet aggregation.

Thus, the objective of this work was to characterize propolis extracts by spectrophotometric and high-performance liquid chromatography (HPLC) methods, and explore influence of propolis on platelet aggregation based on adenosine diphosphate (ADP) induced aggregation testing on the whole blood samples of healthy volunteers.

## Methods

### Materials and propolis extracts

All standards of flavonoids and phenolic acids were obtained from Extrasynthese (Genay, France) and Sigma Aldrich (St. Louis, MO, USA), respectively, and were used as methanolic solutions. Organic solvents were obtained from Fisher Scientific (Hampton, NH, USA) and Kemika (Zagreb, Croatia). Propolis samples were obtained commercially; 19 samples were from Croatia to reflect different composition of propolis between continental and costal part, as well as one from Macedonia and one from Bosnia and Herzegovina for a comparison. Ethanolic extracts of propolis were prepared at room temperature using 80% ethanol (*V/V*) [20]. 1 g of raw propolis was extracted with solvent for one hour, filtered and as such used for chemical characterization. For the purpose of platelet aggregation functional testing extracts were dried using rotary vapor and reconstituted in dimethyl sulfoxide.

### Total polyphenols, flavonoids and phenolic acids

Total content of polyphenols was determined by Folin-Ciocalteu method according to the method described by Slinkard and Singleton [21]. The method is based on the reduction of MoO^4+^ to MoO^3+^ and absorbance is recorded at 765 nm. The results are expressed as equivalents of gallic acid from the calibration curve.

Total flavonoids were determined by Christ-Müller’s method [22]. After acid hydrolysis flavonoids were determined as liberated aglycones spectrophotometrically at 425 nm as a complex with aluminum in a methanol-ethyl acetate-acetic acid medium. Results were expressed as equivalents of quercetin based on calibration curve of quercetin standard.

Total phenolic acids were determined based on European Pharmacopoeia [23] procedure. Phenolic acids form a complex with sodium molybdate-sodium nitrite that has absorbance maximum at 505 nm. Result is expressed as percentage, an equivalent of rosmarinic acid based on formula: Phenolic acids (%) = 0.5 x *A* / *m*, where *A* is measured absorbance and *m* mass of sample used in the analysis.

### HPLC-DAD analysis

HPLC analysis was performed on Agilent 1100 chromatograph equipped with a diode array detector using Agilent Zorbax SBC18 column (250 mm × 4.6 mm, particle size 5 μm) with Zorbax SB-C18 guard column (12.5 mm × 4.6 mm, particle size 5 μm). Mobile phase A consisted of water/methanol/formic acid in volume ratio 93:5:2 and mobile phase B of water/methanol/formic acid in ratio 3:95:2. Flow rate was set to 1 mL/min (volume injected was 10 μL and temperature was set to 40 °C. Following gradient was used: 0 min, 20% mobile phase B, with linear increases to10 min, 40%, 35 min, 50%, 47 min, 50%, 70 min, 80%, followed by re-equilibration to 20% for 10 min. Chromatograms were recorded at 270, 290, 320 and 350 nm. Content of polyphenols was determined using calibration curves of individual flavonoid and phenolic acid [24].

### Platelet aggregation functional test

This study was approved by Ethical committee of the Croatian Institute of Transfusion Medicine (CITM). A total number of 10 volunteers participated in this research, 5 males median age 34 (minimal age 28, maximal age 42) years and 5 females median age 35 (minimal age 28, maximal age 51) years, taking no antiaggregatory medications at least 10 days before giving a blood sample. The main inclusion criteria were: healthy status, age of 18–55 years, no history of thrombosis, known thrombophilia, bleeding diathesis of coagulopathy. These were volunteers that met the legal requirements related to the blood donors’ selection in CITM and were willing to give additional 4.5 mL of blood (calcium citrate used as anticoagulant). Volunteers gave informed written consent.

All blood samples were tested within two hours after collection by treating them with propolis extracts or solvent as a control. Blood samples showed normal aggregation with ADP potentiator (38 to 85 units) measured by Multiplate^®^ (Roche, Switzerland) functional analyzer under the standardized and firmly controlled in vitro conditions in Hemostasis laboratory of CITM. All consumables used in platelet assays were disposed as biological waste under the principles of good laboratory practice and current national regulations (Official Gazette of the Republic of Croatia no. 50/2005).

Platelet aggregation was analyzed by impedance analyzer. General analysis procedure was used: 300 μL of citrate blood was incubated for 3 min with 20 μL of extract solution and 300 μL of calcium chloride saline physiological solution. Analysis was conducted at a temperature of 37 °C. Untreated sample – negative control contained 20 μL of dimethyl sulfoxide. Final concentration of solvent in all experiments was below 3%. Aggregation was initiated by addition of 20 μL of adenosine diphosphate reagent (ADPtest) and was measured for 6 min. Results, expressed as area under curve in arbitrary units (AU), were used for determination of minimal antiaggregatory concentration (*MINaAC*). *MINaAC* represents the lowest concentration of sample that can cause statistically significant reduction of aggregation and was determined as previously reported by our group [15]. Briefly, aggregation of untreated sample (solvent control) and the sample of propolis are measured. If the difference of the recorded aggregation values is greater than 5% the measurement of additional two citrate bloods is performed. Otherwise, double concentration of propolis is taken and the first step is repeated. If the propolis sample in the analyzed concentration shows statistically lower aggregation compared to the untreated sample (assessed by *t*-test) the analyzed concentration is equal to minimal antiaggregatory concentration (*MINaAC*). If not, analysis is performed from the beginning with double the concentration of propolis extract. This presumes that the analyzed concentration is not the first analyzed, else, the analysis is performed from beginning using the half of the concentration of flavonoid.

Difference between means of the control and propolis sample was determined by Student’s *t*-test (at statistical significance α = 0.05) [25]. To be able to use *t*-test, normal distribution of aggregation was assessed using Shapiro-Wilk test (*p* = 0.501) justifying the use of *t*-test [15].

To determine the relationship between *MINaAC* and the content of total flavonoids, phenolic acids, polyphenols, and individual polyphenols, Pearson’s correlation coefficient was calculated as well as its statistical significance (at α = 0.05) assessed using SocSciStatistics online software [25].

## Results

### Chemical characterization of propolis ethanolic extracts

All propolis samples were characterized using spectrophotometric methods on total content of polyphenols, flavonoids and phenolic acids. Individual polyphenols have been determined by liquid chromatography. Results of this spectrophotometry and chromatography analysis are presented in Tables 1 and 2, respectively.

**Table 1:** Content of total polyphenols, flavonoids and phenolic acids determined in propolis extracts by spectrophotometric methods

No	Propolis sample	Polyphenols (mg/g) ^a^	Phenolic Acids (%) ^b^	Flavonoids (mg/g) ^c^
1	Beli Manastir	126.65	16.33	18.17
2	Bjelovar	159.14	25.3	27.5
3	Daruvar	112.11	20.99	20.35
4	Garešnica	142.73	21.24	17.55
5	Gospić	248.01	31.78	6.84
6	Hercegovina	102.65	31.88	14.56
7	Karlovac 1	163.66	17.22	20.69
8	Karlovac 2	277.39	38.67	25.61
9	Kumanovo (FYRM)	62.42	19.86	13.03
10	Lika	155.47	40.71	33.95
11	Lisice (BiH)	128.63	30.3	17
12	Metković	59.23	15.45	9.98
13	Okučani	143.51	37.45	6.83
14	Osijek	153.09	42.77	31.68
15	Pregrada	141.61	8.79	7.35
16	Costal Croatia	72.05	19.56	7.91
17	Sisak	62.09	12.2	9.27
18	Slavonija	176.61	45.67	30.2
19	Varaždin	112.15	30.69	14.55
20	Virovitica 1	142.65	20.45	16.52
21	Virovitica 2	117.16	31.88	55.44

^a^Total polyphenols are expressed as gallic acid equivalents

^b^Total phenolic acids are expressed as percentage of rosmarinic acid equivalents

^c^Total flavonoids are expressed as quercetin equivalents

**Table 2:** Content of individual flavonoids and phenolic acids determined in propolis extracts by liquid chromatography. Results are expressed in mg per gram of propolis sample

		Phenolic Acids				Flavonoids									
No.	Propolis sample	Caffeic Acid	*p*-Coumaric Acid	Ferulic Acid	Isoferulic Acid	Apigenin	Chrysin	Galangin	Kaempferol	Luteolin	Morin	Naringenin	Pinocembrin	Pinocembrin-7- methylether	Quercetin
1	Beli Manastir	2.753	2.347	3.743	2.959	0.751	n.d	12.038	1.281	9.845	n.d.	1.339	1.509	n.d.	n.d.
2	Bjelovar	11.248	11.410	13.616	9.648	1.418	n.d.	27.950	4.604	8.174	n.d.	3.756	4.577	n.d.	1.033
3	Daruvar	2.362	0.931	0.926	n.q.	0.895	11.713	n.d.	n.d.	0.930	n.d.	0.004	n.d.	n.d.	0.460
4	Garešnica	3.524	2.477	1.345	n.q.	0.610	11.628	n.d.	n.d.	0.213	n.d.	0.343	n.d.	n.d.	n.d.
5	Gospić	1.307	11.016	14.704	2.017	0.280	8.224	n.d.	3.501	0.642	0.839	n.q.	1.590	6.128	3.549
6	Hercegovina	0.772	2.838	3.837	1.163	0.371	0.959	n.d.	1.332	0.882	n.d.	0.766	n.d.	n.d.	n.d.
7	Karlovac 1	3.797	9.115	9.581	4.929	0.927	12.891	n.d.	n.d.	2.262	3.126	2.070	1.684	n.d.	2.096
8	Karlovac 2	n.d.	2.037	2.064	0.591	2.679	n.d.	8.151	n.d.	1.195	n.d.	n.d.	n.d.	n.d.	0.577
9	Kumanovo (FYRM)	1.812	0.590	0.177	1.886	0.490	9.090	0.644	0.706	1.616	n.d.	0.896	0.489	4.817	n.d.
10	Lika	8.639	2.076	1.885	5.314	1.571	n.d.	26.600	3.521	6.950	n.d.	3.741	4.033	n.d.	1.225
11	Lisice (BiH)	2.198	3.028	1.065	0.056	1.399	7.844	n.d.	n.d.	0.642	0.004	0.032	0.429	n.d.	n.d.
12	Metković	0.130	0.578	0.522	0.113	0.229	15.468	n.d.	0.245	0.063	n.d.	0.545	n.d.	n.d.	n.d.
13	Okučani	0.780	7.089	8.693	0.793	1.043	n.d.	n.d.	n.d.	1.370	n.d.	0.149	n.d.	n.d.	0.224
14	Osijek	8.332	3.161	1.682	0.306	2.405	n.d.	0.538	n.d.	1.757	n.d.	0.719	n.d.	n.d.	0.724
15	Pregrada	n.q.	1.215	1.544	0.249	0.134	3.905	n.d.	n.d.	1.428	0.730	0.220	n.d.	n.d.	n.d.
16	Costal Croatia	n.q.	2.091	2.649	0.674	0.237	n.d.	4.894	0.827	0.236	n.d.	0.249	n.d.	n.d.	1.430
17	Sisak	0.110	1.201	1.332	0.720	0.310	20.690	1.445	0.438	0.941	n.d.	n.d.	n.d.	n.d.	n.d.
18	Slavonija	6.084	4.241	4.908	4.461	0.868	51.839	1.135	2.049	9.734	n.d.	3.580	n.d.	n.d.	0.548
19	Varaždin	3.304	3.372	3.672	2.347	1.165	47.355	n.d.	3.416	7.837	n.d.	1.410	n.d.	1.697	n.d.
20	Virovitica 1	1.249	5.134	6.310	1.769	0.348	n.d.	7.043	0.912	1.644	0.338	0.794	1.232	2.471	1.217
21	Virovitica 2	5.286	1.499	2.255	n.q.	3.038	6.311	n.d.	7.772	1.606	n.d.	0.076	n.d.	n.d.	0.599

*n.d*. not detected, *n.q*. not quantified

### Antiaggregatory effect of propolis

The effect of propolis extracts on platelet aggregation is shown in Table 3. To include biological variability, minimal antiaggregatory concentration (*MINaAC*) of propolis ethanolic extracts was determined. It includes statistical *t*-test between control and sample and represents concentration of sample that can cause statistically significant decrease of platelet aggregation. As it has previously been reported in μM values, propolis ethanolic extracts concentrations were expressed in molar concentrations taking into account molecular weight of quercetin (*M*_w_ = 302.236). *MINaAC* ranged between 5 μM and 10.4 mM.

**Table 3:** Minimal antiaggregatory concentrations of propolis extracts (*MINaAC*) determined by impedance aggregometry induced by ADP

No	Propolis sample	*MINaAC* (μM)	*p*
1	Beli Manastir	10,432	0.028
2	Bjelovar	326	0.001
3	Daruvar	41	0.002
4	Garešnica	41	0.011
5	Gospić	20	0.029
6	Hercegovina	5	0.034
7	Karlovac 1	163	0.013
8	Karlovac 2	41	0.006
9	Kumanovo (FYRM)	41	0.018
10	Lika	41	0.009
11	Lisice (BiH)	652	0.001
12	Metković	163	0.004
13	Okučani	41	0.001
14	Osijek	326	0.002
15	Pregrada	41	0.001
16	Costal Croatia	20	0.002
17	Sisak	163	0.021
18	Slavonija	41	0.003
19	Varaždin	163	0.002
20	Virovitica 1	81	0.003
21	Virovitica 2	41	0.001

*p*-value determined by Student’s *t*-test

## Discussion

### Chemical characterization of propolis ethanolic extracts

Propolis is extremely rich in polyphenols with content of 59.23 to 277.39 mg of GAE per g of raw propolis (Table 1). This is in accordance with extensive study of ethanolic extracts of propolis collected worldwide by Kumazawa et al. in which quantity of total polyphenols varied between 31 and 299 mg/g also determined by Folin-Ciocalteu method [26].

Content of phenolic acids varies between 8.79 and 45.67% (Table 1). These values are much higher when compared with the results of Kalogeropoulos et al. [27] who attributed phenolic acids to 1 to 9% of total polyphenols. This can be attributed to different methodology of determination GC-MS vs. spectrophotometry as well as fact that spectrophotometric method for determination of phenolic acids in European Pharmacopeia expresses phenolic acids as equivalent of rosmarinic acid (*M*_w_ = 360.32) while most of the phenolic acids found in propolis are much lower molecular weight e.g. caffeic acid (*M*_w_ = 164.05).

Flavonoids ranged between 6.83 and 55.44 mg per gram of propolis (Table 1). As it has been previously shown [26], propolis extracts of different geographical origin have different profile of polyphenols. Generally European propolis has lower quantities of total flavonoids and based on Christ-Müller’s method goes up to 5.5% (propolis sample 21). Ratio of flavonoids in Greek and Cyprus propolis ranged from 4 to 31% of total polyphenols among determined and quantified classes of polyphenolic compounds based on gas chromatography coupled with mass spectrometry analysis [27].

Out of four detected phenolic acids (Table 2) the most abundant was ferulic that was accounted to from 0.2 to 14.7 mg/g (propolis sample 5). This is above values found in Greek and Cyprus propolis (0.1–1.8 mg/g) and lower than those of Polish propolis samples (19.3–44.5 mg/g) [27, 28]. *p-*Coumaric, as well as ferulic, was detected in all samples and content was lower than the one reported for Polish propolis (0.6–11.4 mg/g vs. 37.5–117.0 mg/g) and higher than reported by Barbarić et al. [8] (0.01–0.16 mg/g). Content of caffeic acid was in the range of 0.1 to 11.2 mg/g which is similar to the values observed by Kumazawa et al. [26] (0.2–3.3 mg/g) and Socha et al. [28] (7.7–20.1 mg/g). Isoferulic acid was detected in propolis samples from Serbia [29]. In our study isoferulic acid ranged from 0.1 to 9.6 mg/g.

A total of ten flavonoids have been detected and quantified in propolis ethanolic extracts. Main flavonoids found in all propolis samples were luteolin and apigenin, ranging from 0.1 to 9.8 and 0.1 to 3.0 mg/g, respectively. Both were previously reported as constituents of propolis, and quantities of apigenin are in the range reported for Greek propolis samples (0.25–15.85 mg/g) [27]. Other flavonoids determined in more than a half of analyzed propolis samples were naringenin, kaempferol, chrysin and quercetin with quantities of up to 3.8, 7.8, 51.8 and 3.5 mg/g, respectively. These results are similar to the ones obtained by Socha et al. [28] that determined naringenin, kaempferol, chrysin and quercetin in Polish propolis samples ranging 1.4–21.3 mg/g, 2.4–8.0 mg/g, 13.4–45.5 mg/g and 4.1–7.9 mg/g. Pinocembrin and its 7-methyleter (pinostrobin) were found in eight (0.4–4.6 mg/g) and four (1.7–6.1 mg/g) samples. These concentrations are similar to previously reported values in Croatian propolis; pinocembrin 0.1–7.7 mg/g, and slightly higher for pinostrobin that was reported 0.02–0.25 mg/g [8]. Morin was found in four propolis samples within range of 0.004–3.126 mg/g that is similar in value reported for samples of Chinese propolis of 0.076–0.172 mg/g [30]. Chemical characterization of propolis samples used in this study showed comparable results of Croatian propolis to those reported for propolis samples derived from Eurasia.

When compared to values reported for individual flavonoids of 0.2 to 122 μM in the platelet functional test induced by ADP, propolis shows significant potential to influence platelet aggregation.

As flavonoids are major propolis constituents responsible for pharmacological activity, the mechanism of antiplatelet activity of propolis as well as targeted signaling pathways are the same. Flavonoids inhibit platelet aggregation induced by thromboxane A2, ADP, thrombin and collagen [31]. Underlying mechanisms that prevent platelet aggregation that were reported for individual flavonoids include inhibitions of intracellular signaling pathways through inhibition of the enzymatic activities of phospholipase C [32, 33], phospholipase A2 [34] and cyclooxygenase 1 [35], reducing the oxidative burst and decreasing nitric oxide production [36].

Propolis samples with lowest *MINaAC* values were 5, 6 and 16. Sample 5 had *MINaAC* of 20 μM and it contained pinocembrin-7-methylether as most dominant flavonoid for which *MINaAC* of 0.95 μM was reported [15]. Sample 6 was the most potent in preventing platelet aggregation (*MINaAC* = 5 μM) and it contained luteolin, apigenin and chrysin with *MINaAC* values of 7.6, 3.8 and 3.8 μM [15]. While in samples 5 and 6 comparable values of *MINaAC* between propolis and pure substances was observed, sample 16 contained mainly galangin for which *MINaAC* of 122.17 μM is reported [15]. This could indicate that individual flavonoids can have synergistic effect in propolis enhancing its antiplatelet activity and could be rationalized by different mechanism of action on how individual polyphenol exhibits its biological activity.

When all 21 propolis samples have been taken account and *MINaAC* (Table 3) was related to quantities of individual polyphenol constituents (Table 2), only content of luteolin showed week connection to *MINaAC* that was statistically significant (Pearson’s correlation coefficient 0.4831, *p* = 0.0266). If statistical significance was set to α = 0.1, pinocembrin-7-methyleter would show strong negative correlation to *MINaAC* (*r* = − 0.9146, *p* = 0.0855) [37]. However, this should be considered with caution as pinocembrin-7-methyleter was found only in 4 while luteolin was quantified in all propolis samples. More potent propolis samples have lower *MINaAC* values, consequently samples of propolis with lower content of luteolin and higher content of pinocembrin-7-methyleter showed better antiplatelet activity.

In our preliminary studies we have observed antiplatelet activity of propolis in concentrations of 200 mg/L [18] while Zhang et al. reported 100 mg/L [17] when using ADP as inductor of platelet aggregation. Disadvantage of both studies was testing only arbitrary concentrations of propolis, not a range on which either *IC*_50_ values or *MINaAC* determination can be based of. Moreover, study by Zhang et al. was performed on one propolis sample by optical densitometry assay on platelet rich plasma which can be regarded inadequate as composition of propolis is highly variable [3]. Advantage of this study is that we have analyzed 21 propolis sample of different origin and conducted platelet aggregation assays on whole blood which better reflects in vivo conditions.

Propolis is rich source of polyphenols commonly used as dietary supplement i.e. functional food. Most commonly active components, flavonoids and phenolic acids are extracted with mixtures of ethanol and water in different ratios (optimal 70–80%). This is the first study that shows antiaggregatory potential of propolis ethanolic extracts in low micromolar concentrations on the whole blood that can be attributed to some extent to polyphenolic constituents of propolis in vitro. Results of this study suggest that propolis supplementation can influence function tests of platelet aggregation in vitro. Further studies are needed to confirm beneficial effect in prevention of cardiovascular diseases in vivo.

## Conclusions

Propolis is rich source of polyphenols commonly used as dietary supplement i.e. functional food. Most commonly active components, flavonoids and phenolic acids are extracted with mixtures of ethanol and water in different ratios (optimal 70–80%). This is the first study that shows antiaggregatory potential of propolis ethanolic extracts on the whole blood in the low micromolar concentrations that can be attributed to some extent to polyphenolic constituents of propolis. Results of this study suggest that propolis supplementation can influence function tests of platelet aggregation as well as may have potential beneficial effect in prevention of cardiovascular diseases if proven in human studies.

## Abbreviations

ADP: Adenosine diphosphate
CITM: Croatian Institute of Transfusion Medicine
HPLC: high performance liquid analysis
*MINaAC*: Minimal antiaggregatory concentration
